# Digital paradigm of public health

**DOI:** 10.3389/fpubh.2026.1793593

**Published:** 2026-04-21

**Authors:** Buyandelger Batmunkh, Munguntuul Enkhbat, Erkhes Erdenebaatar

**Affiliations:** 1 Department of Basic Science, Mongolian National University of Medical Sciences, Ulaanbaatar, Mongolia; 2 Department of Public Health Nursing, School of Nursing, Mongolian National University of Medical Sciences, Ulaanbaatar, Mongolia; 3 Independent Researcher, Ulaanbaatar, Mongolia

**Keywords:** big bang model, digital transformation or digital shift of public health, Kuhn cycle, paradigm of public health, paradigm shift and public health

## Abstract

We propose declaring the “Digital Paradigm of Public Health.” To plan the future of public health by focusing on a precision approach within the digital ecosystem, it is necessary to introduce a digital paradigm. The digital revolution has had a profound impact on all fields of health sciences, particularly in the development of public health. In particular, in today’s context, where health has become an accelerator of digital transformation, there is a need to call for a paradigm shift in public health that aligns with this transformation. The transition of the public health paradigm has progressed through naturalist, normative, and functional forms. Following the development of these paradigms, a digital paradigm emerged as the next stage. The digital paradigm of public health is demonstrated through four paradigmatic aspects: ontology, epistemology, axiology, and methodology.

## Introduction

The ‘Digital Paradigm of Public Health’ (DPPH) has been declared. Why? *The Lancet* and *Financial Times Commission* emphasize the need to define an entirely new health paradigm for public health (PH), which will be a critical approach in the future (1). By formulating DPPH, we can create a completely paradigm shift that optimally utilizes advanced digital technologies such as the Internet of Things, artificial intelligence (AI), and machine learning—the outcomes of Industry 5.0 (2)—in the application of PH, thereby guiding the development of PH in the right direction. This is of widespread and critical importance in the 21st century, as health issues rapidly globalize and PH reinvents itself (3). Innovations in digital technologies have a profound impact not only on health, including PH, but also on all sectors of society, thus playing a crucial role in paradigm shifts (4). In recent years, the use of the term ‘digital’ in PH research has increased exponentially, but there is no unified understanding of its exact meaning (5), which we conclude is due to insufficient attention being given to the paradigm shift in PH. In order to clarify the meaning of the term ‘digital’ in PH, we distinguish four analytical levels: ‘digitization’—the conversion of analog information into digital form, such as transforming paper-based records into electronic databases; ‘digitalization’—the use of digital technologies to improve or support existing PH activities, such as electronic reporting, online health communication, and digital surveillance; ‘digital transformation’—a broader process involving fundamental changes in the organization, culture, and delivery of PH services, representing the conceptual core of the proposed DPPH; and ‘artificial intelligence and advanced analytics’—enabling technologies that support predictive modeling and advanced decision-making within digital transformation.

We use the general term ‘DPPH’ throughout this article, the framework is designed to operate primarily at the population and subpopulation levels, while also drawing on insights from individual-level strategies. In this way, DPPH conceptually encompasses approaches aligned with both precision PH, which focuses on predictive, data-driven, and risk-stratified interventions for populations, and personalized PH, which emphasizes tailoring interventions to individual characteristics. By integrating these perspectives, the Digital Paradigm provides a flexible and coherent framework that can guide the application of digital technologies to improve population health, without being restricted to a single analytical level.

While digital technologies are often interpreted as incremental innovations within existing PH frameworks, this study argues that their cumulative effects may represent a deeper conceptual transformation. Therefore, the proposed DPPH is not understood as a purely technological evolution but as a broader shift in how population health is conceptualized and managed.

This transformation includes changes in the underlying assumptions of PH practice, including the transition from retrospective analysis to real-time surveillance, from uniform interventions to risk-stratified strategies, and static population models to dynamic data-driven systems. These developments suggest the possibility of an emerging paradigm transition rather than a simple extension of existing approaches.

A paradigm, as used by Kuhn, is also one of the most important concepts in defining the state of scientific development (6). In the field of PH, paradigm shifts have occurred owing to societal, political, economic, industrial, and technological factors (7, 8). While some scholars conclude that Kuhn’s concept represents a ‘wrong turning’ (9), they do not deny the significance of his paradigm concept, which we must consider when formulating the paradigm of precision PH.

We raised three key research questions that guide the conceptual development of an emerging DPPH: (1) How can a revised interpretive model of scientific progress be formulated, (2) How can this model help to understand ongoing paradigm shifts in PH, and (3) how the conceptual foundations of a DPPH can be articulated.

## Search strategy and selection criteria

For this review, we conducted searches via PubMed, Google Scholar, and PhilPapers with combinations of keywords such as ‘*paradigm of public health*,’ ‘*paradigm shift and public health*,’ and ‘*digital transformation or digital shift of public health*.’ We relied on the articles identified through these searches as the foundation of our review. This study was designed as a conceptual narrative review aimed at supporting the development of a theoretical framework rather than conducting a comprehensive systematic synthesis of empirical evidence. Therefore, the search strategy was intended to identify influential and representative literature relevant to paradigm development and digital transformation in PH. Eligible papers included reviews that addressed the outcomes and future prospects of digital transformation or digital shifts within various scopes of PH. Studies were included if they addressed digital transformation in PH, discussed conceptual or theoretical aspects of PH paradigms, or covered population-level PH systems. Studies were excluded if they focused solely on clinical medicine, addressed individual patient treatment only, or did not discuss PH implications. We restricted our selection to articles published in English in peer-reviewed journals from 2020 onward. This restriction was applied in order to capture recent developments in digital transformation, artificial intelligence in PH, which represent rapidly evolving areas. The COVID-19 pandemic accelerated digital transformation in PH and produced a substantial body of relevant literature, further supporting this time restriction. Earlier foundational literature in philosophy of science and PH theory was included selectively where conceptually necessary. From the search results, we identified and selected articles that encompassed the entire scope of PH rather than focusing on a specific age group or a single component of PH. Additionally, we utilized books and articles that were not identified through the above searches but were relevant to the study. Although a formal systematic review protocol was not followed, efforts were made to ensure transparency by clearly describing the databases searched, keywords used, and selection criteria.

## Rethinking model of scientific progress

The core concepts and approaches focused on Kuhn’s paradigm shift, and more broadly, his view of scientific progress (6, 10) has already evolved to a mature stage (Table 1). However, we support the current need to reinterpret and develop Kuhn’s concept (11). We are currently expanding the use of a new functional approach (12) to explain scientific progress by emphasizing the influence of external factors.

**Table 1:** Basic concepts and approaches of paradigm shift.

Concept/approach	Definition
Prescience	A phase not yet a sufficiently mature conceptual model to address the major problems
Science	A ‘puzzle-solving’, or ‘problem-solving’ process with ‘normal’ and ‘revolutionary’ phases
Normal science	A phase of stable implementation of the process of scientific theory and practice within a particular paradigm
Paradigm	A disciplinary matrix of key theories, instruments, values, and metaphysical assumptions that make up normal science
Anomaly	A phase by which scientific discoveries or situations arise that cannot be explained or resolved within a particular paradigm
Crisis	A phase where the accumulation of anomalies leads to the inability to explain the scientific process according to a certain paradigm
Scientific revolution	A phase to widespread and large-scale advances in science resulting from the competing disagreements (different ideas and preferences)
Paradigm change/shift	A phase of shifting to a new paradigm that cannot be understood through the conceptual framework and terminology of a particular previous paradigm
Kuhn cycle	A model showing the sequence of phases: normal science, anomaly, crisis, revolution, paradigm change
Epistemic approach	This approach defines scientific progress in terms of knowledge
Semantic approach	This approach defines progress in terms of truth or verisimilitude
Functional-internalist approach	This approach construes scientific progress in terms of the function of scientific practice.
New functional approach	This approach defines the development of science by enriching the functional-internalist approach in three ways. For example, qualitative notion of usefulness; not internalist (not only the internal problems of the scientific community); to ‘puzzle-solving’, or ‘problem-solving’, also added problem-defining.

**Figure 1:**
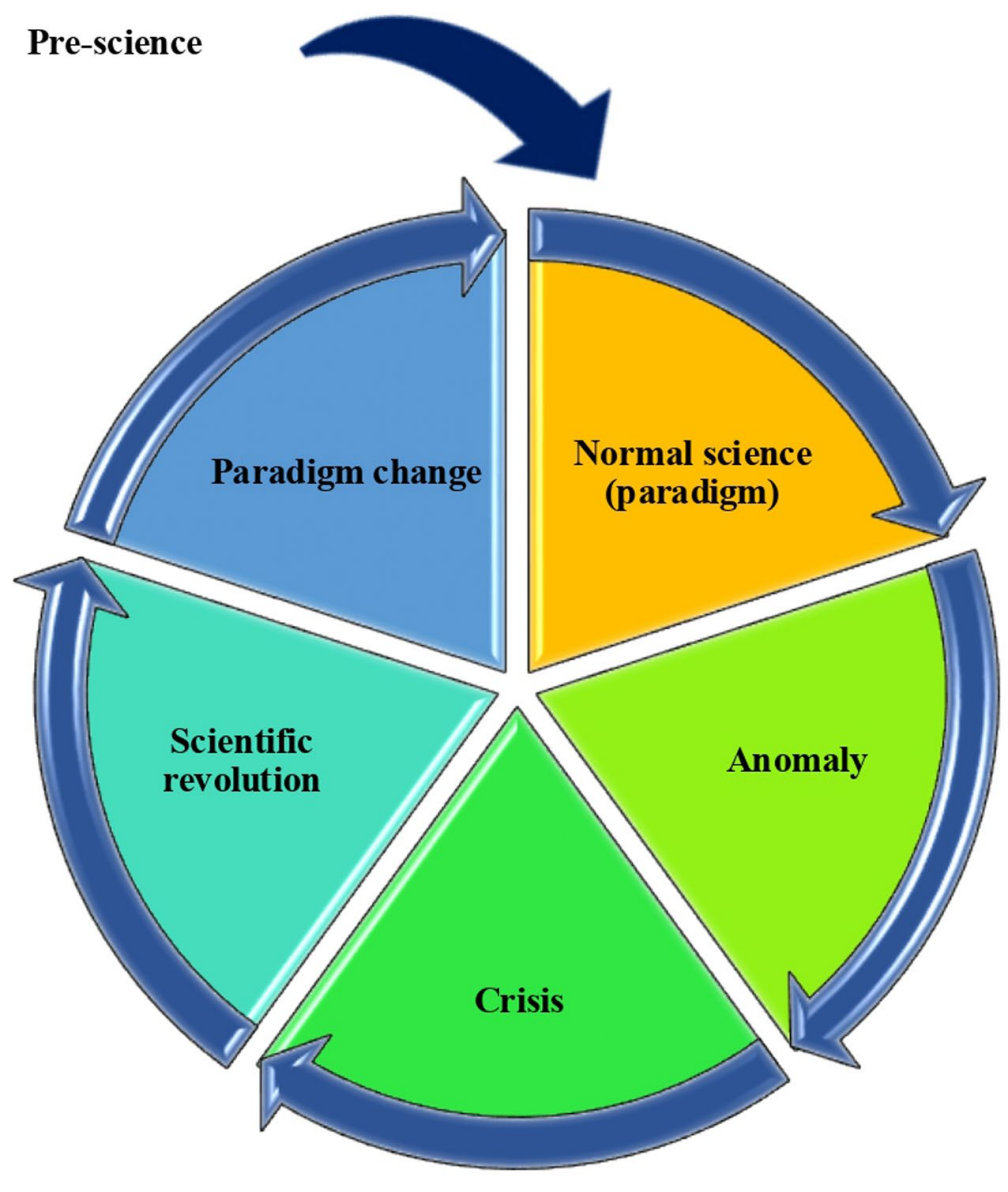
The Kuhn cycle.

The essence of a paradigm shift is ‘change,’ and therefore, its process is influenced not only by internal problems within the scientific field but also strongly affected by external conditions rooted in industrial and technological advancements. This phenomenon has been increasingly recognized in the field of PH (13, 14). When modeling a paradigm shift, expressing it in an *a priori* form often fails to fully capture the actual conditions of scientific progress. The Kuhn cycle (Figure 1) is a representation of *a priori* knowledge that does not account for the influence of space and time on scientific progress, or includes information about external conditions (6).

If we analyze according to the Kuhn cycle, we cannot obtain sufficient information about whether an ‘old paradigm’ existed before our ‘official paradigm,’ how we are connected to those paradigms if they exist, or how a ‘new paradigm’ will form in the future. This leads us to align the paradigm shift process with a general framework of scientific development, which requires us to consider the influence of external conditions alongside spatial and temporal factors.

**Figure 2:**
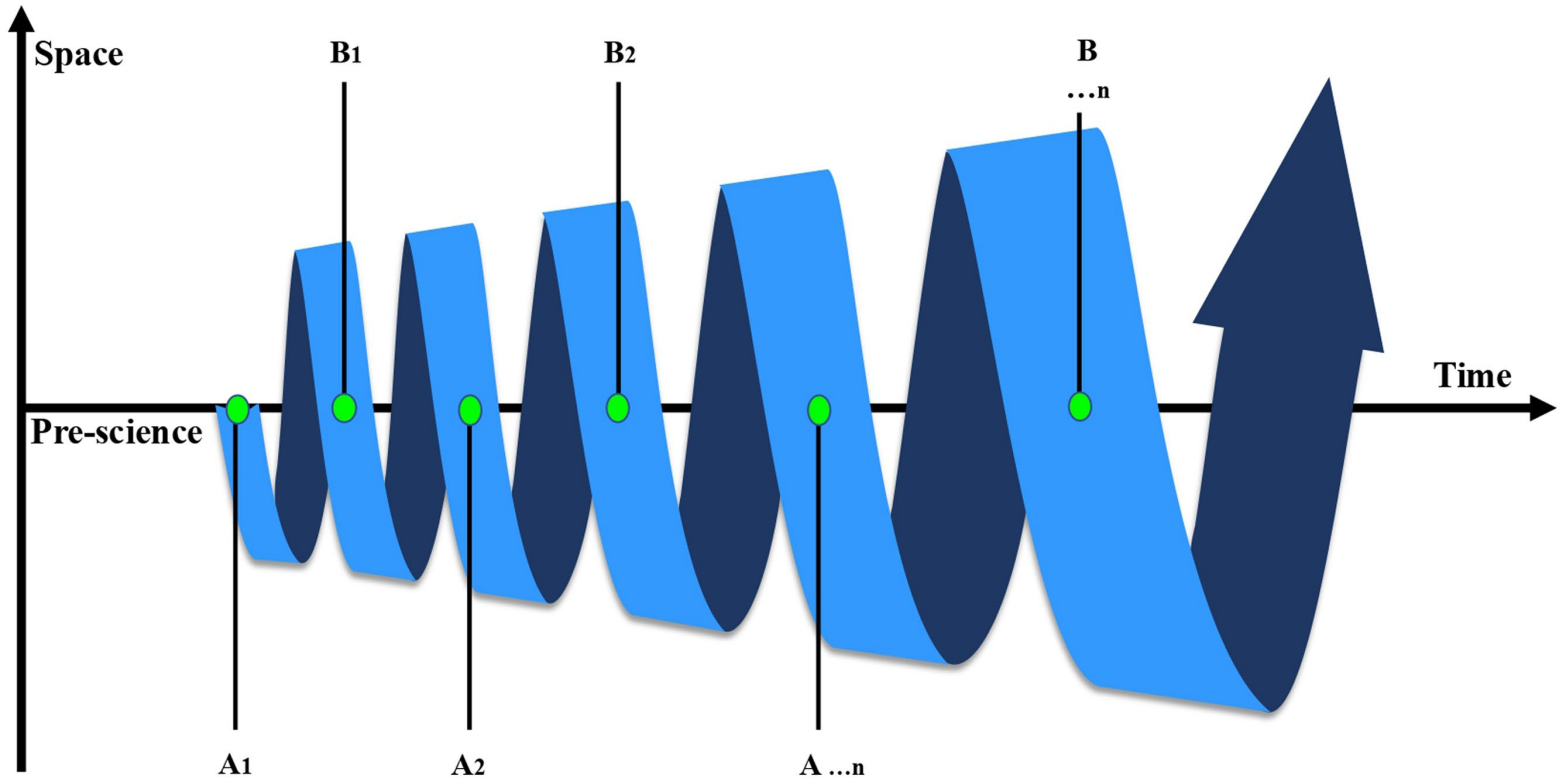
Big bang model of scientific progress. External conditions such as production, technology, and culture develop according to the same regularity as this spiral. As science advances, these factors gradually enter the domain while simultaneously expanding their scope by reflecting scientific influence, driving continuous development over time. The Kuhn cycle progresses in a circular manner through points Al-B1→B1-A2→A2-B2→B2-A...n→A...n-B...n, driving paradigm shifts.

It is possible to develop and expand a spiral model of *a posteriori* knowledge that incorporates several Kuhn cycles. This approach does not challenge the ‘epistemic virtue’ (15) of a specific scientific field but instead provides a way to view scientific development within a space influenced by industrial, technological, and cultural factors. When viewed in conjunction with Kuhn’s (6) historical study of scientific development, our proposed model is more closely related to the concept of ‘big history’ (16) in terms of content. In this context, we do not equate the history of science with the history of the universe but rather highlight that the development of science, at a certain stage, follows similar patterns of evolution. In the field of health sciences, this shift in focus toward a broader space is evident in initiatives such as the ‘One Health’ (17) approach and the ‘Planetary Health’ initiative (18). These developments are creating conditions that guide the advancement of PH within a wider framework. Based on these foundations, we propose aligning scientific processes with the standard model of cosmology, the Big Bang Model (19), and mapping paradigm shifts via the ‘Big Bang Model of Scientific Progress’ (Figure 2).

The ‘Big Bang Model of Scientific Progress’ does not alter the Kuhn cycle but incorporates it into a continuous and ongoing process (Table 2). This allows for a clearer explanation of Kuhn’s rationale that ‘two paradigms can coexist peacefully’ (6). As long as they retain sufficient theoretical and empirical validity, old paradigms can coexist (and even enrich themselves) within the context of newer paradigms by evolving and adapting appropriately.

**Table 2:** Comparison between Kuhn cycle and big bang model.

Feature	Kuhn cycle	Big bang model
Structure	Cyclical	Expanding spiral
Focus	Internal scientific crises	Internal, and external forces
Time dimension	Discrete phases	Continuous evolution
Paradigm coexistence	Limited	Central feature
Policy relevance	Low	Higher

The Big Bang Model of Scientific Progress proposed in this study is intended primarily as a heuristic framework rather than a literal or predictive theory of scientific change. The cosmological metaphor is used to illustrate the possibility that scientific development may occur through periods of rapid conceptual expansion, followed by phases of consolidation and stabilization. The use of cosmological metaphors in this article is intended as an analytical tool to describe long-term processes of scientific development rather than as a literal analogy. The Big Bang metaphor allows the visualization of scientific paradigms as evolving within an expanding space shaped by technological, social, and intellectual factors. In contrast to linear or cyclical models, this perspective emphasizes the coexistence and gradual transformation of paradigms over time.

In this interpretation, the metaphor serves as an analytical tool for understanding how new clusters of ideas, methods, and technologies may emerge and interact to produce periods of accelerated transformation. The model is therefore not meant to replace existing theories of scientific progress but to complement them by emphasizing the dynamic and multidirectional character of contemporary PH development.

While classical paradigm theory emphasizes discontinuous scientific revolutions, the Big Bang Model highlights the possibility of overlapping and interacting waves of innovation. The model should therefore be interpreted as a conceptual tool rather than a definitive theory of scientific development.

## Paradigm shift in public health

**Figure 3:**
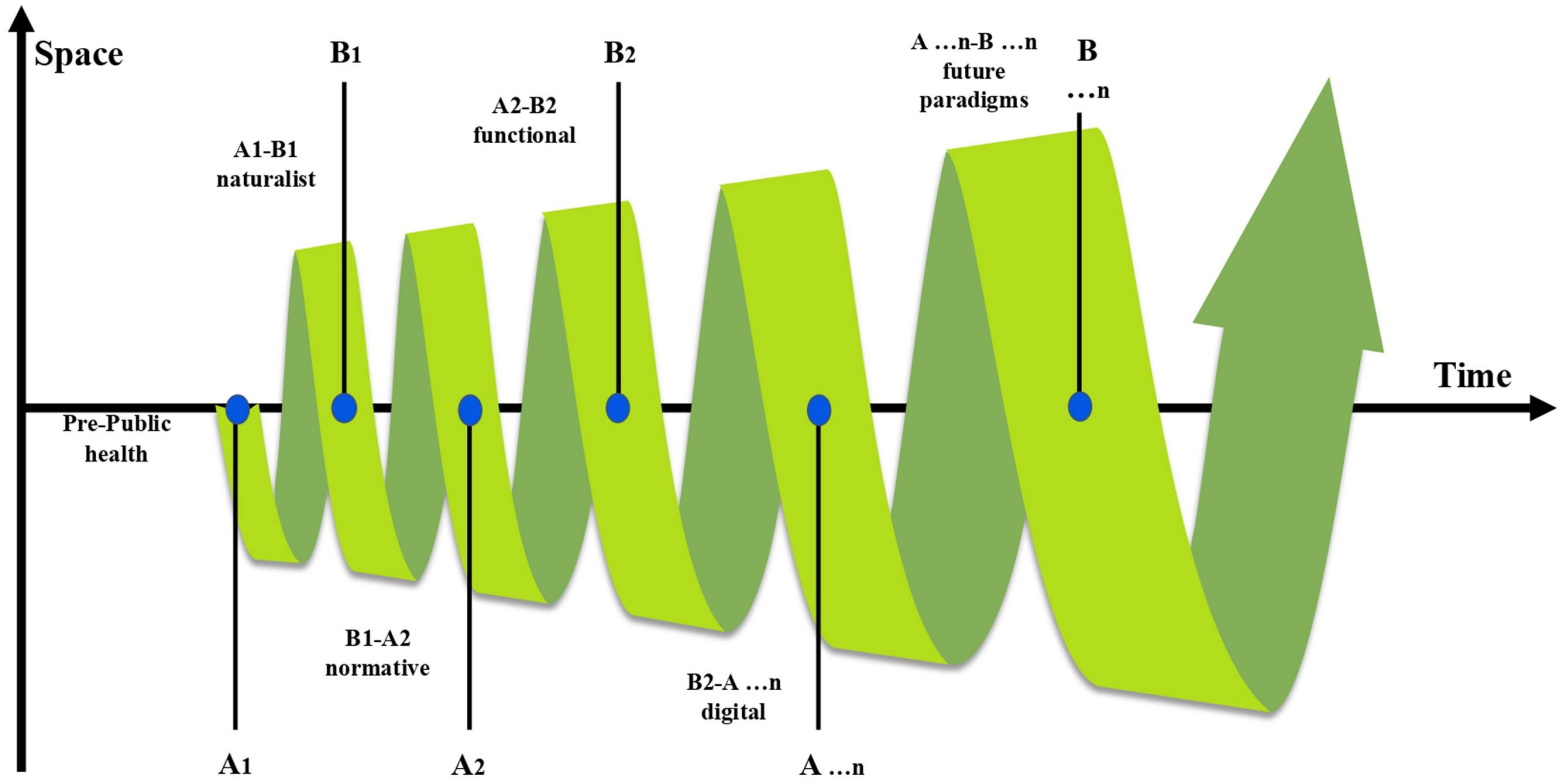
Model for the paradigm shift of public health.

According to researchers, the prevailing view defines PH as science and art (20). From the standpoint of our proposed model, we argue that the PH, as a science, not only evolves and transforms but also constitutes a paradigmatic system of knowledge shaped by distinct philosophies, methodologies, and tools influenced by space and time. Researchers have indicated that discussions of paradigm shifts in PH have focused primarily on specific aspects of the field (14, 21, 22). However, as we are seeking a paradigm shift that can define the development of PH as a whole, these studies do not provide sufficient answers. Therefore, we summarize the paradigm shift in PH using the ‘Model for the Paradigm Shift of Public Health’ (Figure 3).

We define paradigms that can comprehensively encompass the scope of PH in the broadest sense based on three distinct philosophical perspectives: naturalist, normative, and functional. These paradigms focus on the unique challenges of PH. The naturalist paradigm of PH is grounded in the philosophical view that defines health as the absence of disease (23, 24). This paradigm has played a crucial role in establishing the legitimacy of the scientific systems within PHs and shaping their outcomes (25). During the development of the naturalist paradigm, epidemiology, the basic science of PH, has formed a solid foundation through close integration with statistics, microbiology, engineering sciences, and biomedical and environmental sciences (26). This has led to significant achievements in areas essential for human health, such as infrastructure and infectious disease control (27). Subsequently, theoretical advancements such as the ‘epidemiological transition’ (28) shifted focus toward chronic diseases, highlighting the naturalist paradigm’s characteristic disease-centered approach. The naturalist paradigm continues to undergo renewal to this day and can be observed in the continuous development of new epidemiological methodologies (29, 30).

From a normative perspective, determining the condition of a disease is not merely about uncovering natural laws but also emphasizes the importance of considering its inseparable connection to behavior (or feeling) and certain social norms (31, 32). Based on these ideas, disease was viewed not only as an individual concern but also as a societal problem, laying a deeper foundation for PH services centered around medical institutions (33, 34). The population-centered approach, a core node of this paradigm, not only directed PH toward focusing on healthy lifestyles but also led to a more detailed examination of disease risk factors. This has spurred the rapid development of disciplines such as occupational and environmental health, contributing immeasurably to the progress of population health (35). While the normative perspective aligns with the achievements of naturalism in many ways and shapes PH’s ‘best practices,’ (36) normative views on health continue to evolve (37).

Simultaneously, holistic perspectives have sought to overcome the limitations of both naturalist and normative approaches (38, 39). Holistic proponents are grounded in two key principles: (a) value criterion and (b) explanatory criterion (40). Although the holistic perspective did not develop into an independent paradigm of PH, it laid an important ideological foundation for the emergence of subsequent paradigms by asserting that health cannot be fully defined in a value-free manner (41).

We define the next paradigm of PH as a functional paradigm with a behavior-centered approach. The key common understanding of the previously mentioned concepts of PH is the organism’s function from a comparativist perspective (42). Functionalists formulate this concept at a high level, emphasizing two general functions—precision and variation—both grounded in the common features of natural and value-laden (biological, social, and behavioral) functions (43). Viewing health through the lens of functions leads to the conclusion that the prolonged continuation of improper behaviors, such as smoking, excessive alcohol consumption, and unhealthy diets, impairs life functions and results in unhealthy states (44). One major manifestation of the many advances in the field of PH within the functional paradigm is the philosophical approach called ‘new public health (NPH)’. The NPH is an action-based approach in which population and individual health are considered equal issues within the framework of environmental, political governance, social, and economic development (45).

The next shift in the paradigm of PH undoubtedly takes shape on the foundation of understanding health in the context of comprehensive societal development, fully incorporating the influences of paradigm shifts in other fields. For example, the paradigm shift in pandemic research due to COVID-19 is moving ‘from responsive to proactive or preemptive’ (46), whereas the current paradigm shift in medicine is characterized by a shift ‘from real-time diagnostics and treatment to prediction and prevention’ (47). Ongoing digital and genomic revolutions (48) are impacting health, healthcare, living, and society on a wide scale. Therefore, we propose defining the next paradigm of PH as the ‘digital paradigm.’ This paradigm can provide the most suitable foundation for formulating and establishing the necessary elements to build a governance architecture for PH science based on the precision PH approach (1).

## Digital paradigm of public health: conceptual foundations and operational implications

**Figure 4:**
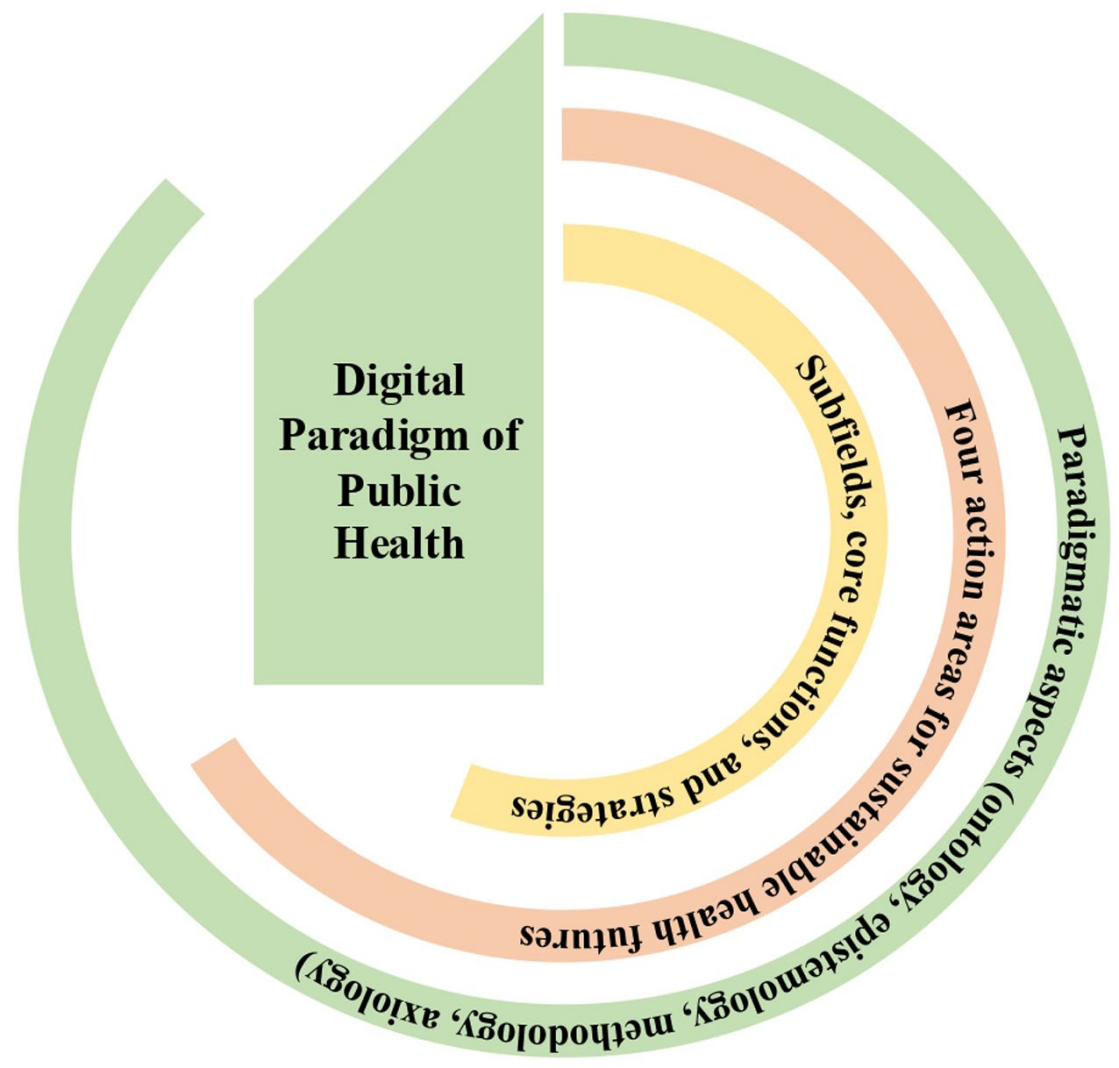
Outline between paradigmatic aspects and action areas of the digital paradigm.

A paradigm is fundamentally composed of four paradigmatic aspects that are intrinsically linked to its essence: ontology, epistemology, methodology, and axiology (49). The *four action areas for sustainable health futures* (1)are grounded in the four paradigmatic aspects of the DPPH (Figure 4). From the perspective of our proposed ‘Big Bang Model of Scientific Progress,’ the DPPH represents the most recent developmental stage of PH, extending into a broader space than previous paradigms did. Therefore, the causative concept that disease is not solely a nonbiological phenomenon but also depends on the interactions of adjacent causal processes, such as behavioral, social, environmental, and technological factors—continuously interacting with humans—should form the philosophical foundation of the DPPH.

Although the DPPH is primarily proposed as a conceptual framework, its practical relevance depends on the possibility of operational implementation. The value of a paradigm ultimately lies in its capacity to guide transformation in PH systems. Operationalizing the DPPH therefore requires the development of practical pathways linking the four paradigmatic aspects—ontology, epistemology, methodology, and axiology—to PH practice.

### Ontological aspects

When considering ontology in the field of science, four criteria are taken into account: (1) developed to be common resources, (2) developed and validated by domain experts, (3) recognized as being subject to further development, and (4) independent of format and implementation (50). Digital technologies have rapidly spread across all sectors of society because the waves of the Fourth and Fifth Industrial Revolutions have become the foundational ontologies for digital transformations in the public health sector. This ontology has a wide range of forms and applications, such as digital epidemiological surveillance, rapid case identification, interruption of community transmission, public communication, clinical care, tiered telementoring, telecritical care, robotics, and AI for monitoring (51, 52). Moving forward, this ontology is expected to deepen. Currently, some technologies, such as blockchain, which are in the conceptual stage or early development phase in the health sector, are likely to overcome their challenges and advance toward greater maturity (53, 54).

Operationally, the digital paradigm may be operationalized through the integration of diverse digital data sources, including electronic health records, environmental monitoring systems, and digital epidemiological surveillance platforms. The consolidation of interoperable data infrastructures has been identified as a critical prerequisite for modern PH intelligence systems (55, 56). Empirical studies on PH further demonstrate that integrated data architectures enable risk stratification and the identification of vulnerable population subgroups with greater granularity (57, 58).

### Epistemological aspects

From an epistemological perspective, we examine the content of good reasoning and logic within the framework of the digital paradigm (59). In this context, it is reasonable to focus on evidence-based critical examination rather than true or false beliefs (60). Currently, researchers in the field of PH interpret key concepts such as digitization, digitalization, and digital transformations differently (1, 5). Based on this evidence, a conceptual framework with logical arguments forms the basis for declaring the digital paradigm. However, there is a growing need to develop unified definitions of categories used in precision PH. Additionally, one of the challenges in the field of PH epistemology in the coming years is combating misinformation that may arise during efforts to improve communication between health professionals and patients. Notably, attention must be paid to communication skills when digital tools are used (61). Thus, the expansion of digital epistemology in the field of PH is becoming crucial in shaping this new paradigm.

The digital paradigm reconfigures knowledge production toward data-driven and predictive modes of reasoning. Real-time surveillance systems, predictive modeling, and machine learning–assisted analytics have been shown to support earlier detection of emerging threats and enhance anticipatory response capacities, particularly during pandemics (62–64). Such approaches shift PH from predominantly retrospective assessment toward prospective risk forecasting.

### Methodological aspects

Evaluating scientific outcomes solely on the basis of scientific knowledge is insufficient, as ‘methodologically, the most important aspect of science is the specific way in which it investigates its object’ (65). Scientific methods based on digital technologies have already been implemented in the field of PH. For example, digital health dashboards are essential not only for enhancing public health policy decision-making but also as a ‘a paradigm-changing approach to addressing future epidemics and pandemics’ (66). Furthermore, it is worth noting that methodological digital developments such as NLP (natural language processing), ‘DigiPHrame’ (a comprehensive framework for the development and assessment of digital technologies designed for public health purposes), and HIT (health information technology) are being actively pursued (67–69). In addition, efforts are being made to improve the application of generative pretraining transformers (GPTs). Because the GPT does not sufficiently express causality in health-related issues, a ‘conceptual map’ for fine-tuning it has been proposed (14, 70). AI tools contribute methodologically in multiple ways, such as significantly enhancing the precision of health education, management, and operations targeted at chronic diseases (71–73). However, there is an urgent need to address the current lack of methodologies to determine which digital technologies are most suitable for which target groups in health areas. Consequently, PH organizations must carefully develop strategies to effectively utilize AI to fulfill PH responsibilities (74, 75).

Operationalization requires the institutional embedding of digital decision-support tools, integrated dashboards, and interoperable health information systems within routine PH practice. Emerging global initiatives emphasize that digital transformation in health systems requires coordinated governance mechanisms, interoperability standards, and institutional alignment to translate technological innovation into a system-level impact (76). A recent scoping review highlighted that the future trajectory of precision PH is closely linked to the practical embedding of predictive analytics, real-time surveillance, and integrated health information systems within routine PH decision-making processes (77). However, the effectiveness of implementation depends on interoperability standards, workforce competencies, and sustainable financing models.

### Axiological aspects

In the realm of scientific axiology, ethics is considered foremost (78). From this perspective, it has been emphasized that the digital transformation within a PH’s digital ecosystem must be rapid and successful and that the development of its unified purpose and vision is critically important. This highlights the formation of the axiological aspect of the digital paradigm in its most general sense (79). Although digital technologies in the health sector are rapidly advancing, safeguarding privacy and ensuring ethical deployment remain among the most crucial issues (80). The use of AI raises several challenges, such as addressing potential health inequities in PH policies and practices, identifying the limitations and risks of AI better, and ensuring standardization (81, 82). Moreover, there are even proposals to remove digital tools from the healthcare market if they fail to meet the minimum requirements for privacy, security, and ethics (83). This underscores the axiological foundation necessary for the existence of a digital paradigm.

A digital paradigm requires robust regulatory and governance frameworks to safeguard vulnerable populations. Recent policy analyses highlight significant gaps in the regulatory mechanisms governing digital environments for children and adolescents, underscoring the need for stronger PH-oriented digital governance structures (84). International guidance emphasizes that digital PH transformation must be accompanied by safeguards addressing privacy protection, algorithmic transparency, equity considerations, and accountability in AI deployment (85, 86). Without such normative guardrails, digitalization risks reinforcing structural inequities or enabling forms of technological determinism. The digital divide, particularly in low- and middle-income settings, remains a significant constraint, with uneven infrastructure, connectivity, and institutional capacity potentially limiting equitable implementation (87).

### System preparedness and future health challenges

In addition to system modernization, DPPH may strengthen preparedness for emerging global health challenges, including future pandemics, climate-sensitive health risks, demographic transitions, and persistent health inequities. Digitally enabled surveillance networks, early warning systems, and integrated One Health data platforms have been increasingly recognized as essential components of resilient health systems (17, 18, 88).

Operationalization also has significant governance and workforce implications. An effective digital PH requires interoperable data-sharing agreements, regulatory oversight mechanisms, and cross-sector coordination capacity. Concurrently, workforce transformation is necessary with expanded competencies in digital epidemiology, data science, systems evaluation, and ethical AI governance (89, 90). Capacity-building efforts must be context-sensitive and adaptable across diverse economic settings.

Possible indicators for evaluating the development of the digital paradigm may include: the degree of digital data integration in PH systems; use of predictive analytics in PH decision-making; coverage of digital surveillance systems; equity of access to digital PH services; and implementation of ethical and regulatory frameworks for digital health technologies.

These elements provide a preliminary basis for operationalizing the DPPH and may guide future empirical research. Further work is needed to refine evaluation frameworks and develop practical tools for assessing digital readiness and impact within PH systems.

## Limitations and critical considerations of the digital paradigm

While the DPPH offers important opportunities for advancing population health, its implementation also raises significant challenges and limitations. A balanced assessment is necessary to avoid overly optimistic or technodeterministic assumptions about the role of digital technologies in PH.

### Digital divide and risks of exclusion

Unequal access to digital technologies can exacerbate health inequality. Populations with limited access to digital infrastructure, lower digital literacy, or reduced economic resources may benefit less from digitally enabled PH services. If not carefully managed, digital transformation may unintentionally exclude vulnerable populations, thereby reinforcing rather than reducing the health disparities. Therefore, PH strategies must prioritize accessibility and inclusiveness to ensure that population-targeted interventions benefit diverse groups.

### Algorithmic bias and data inequities

The increasing use of AI and machine learning in PH introduces new ethical and methodological challenges. Algorithms trained on incomplete or unrepresentative datasets may produce biased results that disproportionately affect certain populations. Additionally, the unequal global distribution of health data resources can create asymmetries in knowledge production, particularly between high-income and low- and middle-income countries. Transparent governance structures and equitable data-sharing practices are necessary to mitigate these risks.

### Risks of technological determinism

Digital technologies should be understood as tools that support PH objectives rather than as solutions in themselves. Overemphasizing technological interventions may divert attention from structural determinants of health, such as socioeconomic conditions, education, and environmental exposures. The DPPH should therefore be interpreted as an integrative framework that complements, rather than replaces, existing PH approaches.

### Infrastructure and resource constraints

The successful implementation of digital PH systems depends on robust information and communication technology infrastructure, reliable connectivity, and technical capacity. In many resource-limited settings, these prerequisites may be insufficient, thereby limiting the practical impact of digital interventions. The development of the digital paradigm should include flexible approaches that can be adapted to varying levels of technological readiness.

### Sociotechnical and governance challenges

The adoption of digital tools is shaped by social, cultural, and organizational factors. Workforce skills gaps, institutional inertia, and regulatory complexity may limit the effectiveness of digital transformation. Effective deployment requires transparent governance frameworks that address data privacy, interoperability, accountability, and responsible AI use. Without such structures, public trust and adoption may be compromised.

### Axiological dimension: ethics, equity, and governance

The axiological foundation of DPPH is expanded beyond general ethics to address operational realities, including the following: (1) digital equity: ensuring accessible digital PH tools for all populations to prevent widening disparities; (2) data governance: transparent, accountable, and interoperable management of health data; (3) regulatory frameworks: policies and standards protecting privacy, security, and ethical use of AI. Integrating these considerations ensures that the ethical, governance, and equity aspects of the digital paradigm are actionable and responsive to real-world PH contexts.

### Conceptual limitations and operational considerations

As a developing framework, DPPH remains primarily conceptual. Empirical validation and operational refinement are needed to evaluate its applicability in diverse PH contexts. Potential measurable indicators may include the following: (1) the degree of digital data integration in PH systems; (2) coverage of predictive analytics in decision-making; (3) population subgroups reached by targeted interventions; (4) implementation of governance or ethical frameworks for digital health technologies. Applied examples, such as digital dashboards for real-time surveillance or AI-supported risk stratification, illustrate how the paradigm could be operationalized. Integrating critical perspectives on equity, ethics, infrastructure, and governance strengthens the conceptual rigor of the paradigm and ensures its relevance for real-world PH systems.

## Conclusion

Thus, the formation of the four paradigmatic aspects of DPPH can serve as a robust scientific foundation to drive the development of the *Four Action Areas for Sustainable Health Futures*. Hybrid theories in PH clearly emerge within the framework of DPPH. This will significantly influence the renewal of PH subfields (such as preventive and population healthcare, occupational safety and health, health economics, biostatistics, epidemiology, and environmental health, etc.), core functions (assessment, development, and assurance), and strategies (surveillance, screenings, awareness, air quality, and regulations, etc.) in the new digital era.
